# Neural Radiance Fields: Driven Exploration of Visual Communication and Spatial Interaction Design for Immersive Digital Installations

**DOI:** 10.3390/jimaging11110411

**Published:** 2025-11-13

**Authors:** Wanshu Li, Yuanhui Hu

**Affiliations:** College of Fine Arts, Jiangxi Normal University, Nanchang 330022, China

**Keywords:** neural radiance fields, instant neural graphics primitives, lightweight modeling, multimodal perception, real-time rendering, immersive interaction

## Abstract

In immersive digital devices, high environmental complexity can lead to rendering delays and loss of interactive details, resulting in a fragmented experience. This paper proposes a lightweight NeRF (Neural Radiance Fields) modeling and multimodal perception fusion method. First, a sparse hash code is constructed based on Instant-NGP (Instant Neural Graphics Primitives) to accelerate scene radiance field generation. Second, parameter distillation and channel pruning are used to reduce the model’s size and reduce computational overheads. Next, multimodal data from a depth camera and an IMU (Inertial Measurement Unit) is fused, and Kalman filtering is used to improve pose tracking accuracy. Finally, the optimized NeRF model is integrated into the Unity engine, utilizing custom shaders and asynchronous rendering to achieve low-latency viewpoint responsiveness. Experiments show that the file size of this method in high-complexity scenes is only 79.5 MB ± 5.3 MB, and the first loading time is only 2.9 s ± 0.4 s, effectively reducing rendering latency. The SSIM is 0.951 ± 0.016 at 1.5 m/s, and the GME is 7.68 ± 0.15 at 1.5 m/s. It can stably restore texture details and edge sharpness under dynamic viewing angles. In scenarios that support 3–5 people interacting simultaneously, the average interaction response delay is only 16.3 ms, and the average jitter error is controlled at 0.12°, significantly improving spatial interaction performance. In conclusion, this study provides effective technical solutions for high-quality immersive interaction in complex public scenarios. Future work will explore the framework’s adaptability in larger-scale dynamic environments and further optimize the network synchronization mechanism for multi-user concurrency.

## 1. Introduction

Contemporary immersive digital installations are gradually reshaping the perceptual boundaries between people and space. Their core lies in constructing a multidimensional experience field with realistic vision, immediate response, and natural interaction [1,2]. In museums, art exhibitions, and urban public spaces, audiences no longer accept static viewing; they expect visual feedback tightly synchronized with their bodily movements [3]. This type of system needs to continuously output high-fidelity three-dimensional images under complex lighting, multiple concurrent users, and free movement conditions to form a spatial narrative that blends the real and the virtual [4,5,6]. However, achieving continuous, stable, and depth-based visual presentation places stringent demands on modeling accuracy, computational efficiency, and perceptual responsiveness [7,8]. Installation design must transcend the limitations of a single medium by integrating spatial perception, real-time rendering, and dynamic interactive logic. This integration constructs an intelligent visual architecture with environmental adaptability and behavioral responsiveness, giving digital content a perceptible, accessible, and evolvable spatial presence.

Current immersive digital devices face multiple technical coupling challenges in achieving the integration of high-fidelity three-dimensional vision and dynamic spatial interaction. The system needs to continuously generate image sequences with realistic lighting, fine textures, and continuous perspective transitions within limited computing resources [9,10]. However, existing rendering paradigms are prone to frame rate fluctuations as scene complexity increases, affecting visual smoothness [11]. When users rapidly move or rotate, if the perspective update fails to precisely match the rhythm of the movement, it can cause perceptual dislocation and weaken the sense of spatial immersion [12,13,14]. Multi-user concurrent scenarios further increase system load, and the parallel computing requirements of different perspective paths place higher demands on model distribution and rendering scheduling [15,16]. Dynamic lighting changes further challenge visual consistency: under low illumination or strong backlight, reconstructed data tends to accumulate noise [17]. In addition, device deployment is often limited by storage and loading efficiency. High-precision 3D models often come with large file sizes, which prolong startup time and are not conducive to rapid on-site configuration and iteration [18]. The pose tracking module is susceptible to sensor noise during high-frequency motion, causing small but noticeable visual jitter and destabilizing the image. More critically, the lack of a dynamic adaptation mechanism between visual generation and motion prediction makes it difficult to adjust the rendering strategy based on movement speed, resulting in motion blur or prediction errors during close-range, rapid motion [19,20]. These factors jointly restrict the long-term stable operation of the device and the consistency of user experience in real public spaces. There is an urgent need for a technical path that can achieve coordinated optimization between modeling density, perception accuracy, and rendering efficiency to support the sustainable output of high-quality immersive experiences in complex environments.

This paper aims to build a NeRF-driven immersive interactive system for real-world deployment. Its core goal is to achieve the coordinated optimization of high visual fidelity and low system latency. The study proposes a comprehensive lightweight modeling paradigm based on Instant-NGP, incorporating sparse hash coding and parameter distillation to significantly reduce model size and inference overhead without compromising radiance field expressiveness. On this basis, a multimodal pose tracking module is designed, fusing depth camera and IMU data and incorporating Kalman filtering to improve motion estimation accuracy, particularly for maintaining stable tracking under rapid rotation and occlusion conditions. The system further integrates the NeRF model with the Unity engine, develops a dedicated shader to handle implicit field queries, and employs an asynchronous rendering strategy to decouple viewpoint prediction and image generation, effectively reducing response latency. The entire architecture forms a closed-loop driving mechanism of “efficient modeling, accurate perception, and low-latency rendering,” enabling seamless multi-user interaction in complex lighting and high-dynamic motion scenarios. Compared to existing methods, this work not only improves frame rate stability and visual continuity but also achieves systematic breakthroughs in model loading efficiency, interaction jitter suppression, and motion adaptability. The paper’s primary innovation lies not merely in its choice of technical modules, but in its unique system integration paradigm. At the model lightweighting stage, we move beyond isolated pruning or quantization. Instead, we propose a joint “targeted extraction–frozen protection” optimization strategy: structured channel pruning is guided by weight contribution analysis from key viewpoints, while a novel high-frequency weight protection mechanism ensures that texture and edge details remain intact after compression. In the perception-rendering closed loop, we designed a multi-modal fusion architecture based on Kalman filtering, aligning its output with the asynchronous rendering pipeline in Unity engine for sub-millisecond synchronization from physical motion to visual feedback, effectively suppressing jitter errors under high dynamic interaction. Finally, we embedded lightweight NeRF models natively into Unity via GPU, constructing an efficient “query-compute-display” pipeline through custom shaders and dual-buffer CUDA kernel functions, solving interoperability challenges between implicit models and mainstream graphics engines. These synergistic designs form an organic whole, with their combined benefits (low latency, compact size, high stability) far surpassing the sum of individual module optimizations, demonstrating substantial contributions to system engineering at this level.

## 2. Related Work

In recent years, the technology approaches for 3D scene representation and real-time rendering have diversified. Multi-View Stereo (MVS) relies on image matching to generate dense point clouds. While it offers fast reconstruction speed, it is prone to producing holes in texture-missing areas, and the rendering quality is limited by the sampling density [21,22]. Voxel grids use regular spatial divisions to facilitate GPU (Graphics Processing Unit) acceleration, but memory consumption increases exponentially with resolution, making it difficult to support fine structures [23,24]. Point cloud rendering directly visualizes collected data and has the advantage of being lightweight, but it lacks surface continuity and occlusion reasoning capabilities. Three-dimensional Gaussian Splatting, an emerging explicit representation method, achieves high-quality real-time rendering through differentiable rendering, but still suffers from blurring and drift in dynamic lighting adaptability and long-distance viewpoint extrapolation [25,26]. While the original NeRF demonstrates excellent visual quality, it requires several seconds to render each frame, completely failing to meet interactive requirements [27,28]. To alleviate this latency issue, Instant-NGP began to attract attention. Its hash coding structure significantly shortened the training and inference cycles, making it the mainstream infrastructure for current acceleration models [29]. Xin C proposed a 3D reconstruction method for soybean plants in the field based on smartphone video acquisition, adaptive frame extraction, motion recovery structure and instant neural graphics primitives (Instant-NGP). While ensuring high-precision reconstruction quality, it significantly improved efficiency and reduced costs, providing an efficient and feasible technical solution for agricultural information breeding [30]. However, most solutions remain at the prototype verification stage and lack deep coupling mechanisms with mainstream graphics engines, resulting in the fragmentation of the rendering pipeline and the accumulation of scheduling delays. Overall, although various methods have made progress in specific dimensions, there are still obvious shortcomings in comprehensive indicators such as complex lighting adaptation, model compactness, and multi-user low-latency response, and a closed-loop interaction system suitable for deployment in real scenarios has not yet been formed.

To address the limitations of 3D scene modeling in terms of real-time performance and deployment feasibility, previous research has attempted to optimize it through model compression and perception enhancement. Some work has adopted knowledge distillation strategies to migrate model behavior to a small network to accelerate inference, but this often results in the loss of high-frequency details [31,32]. Channel pruning techniques reduce computational complexity by sparsifying network activation paths, thereby reducing parameter size while maintaining a certain level of fidelity [33]. At the system integration level, Unity and Unreal engines have gradually supported custom shaders and asynchronous rendering queues, providing underlying support for efficient rendering of non-standard geometry types [34]. Tsai Y T designed a baseball pitching training system that combines the Unity engine and a digital glove. It achieves virtual interaction through tactile feedback and motion capture. Experiments have shown that it can effectively provide physical feedback and has application potential in medical rehabilitation and sports training. The system realizes seamless data interaction between virtual scenes and hardware devices [35]. However, these attempts have mostly been isolated improvements, lacking end-to-end collaborative optimization: model compression has not been designed in conjunction with rendering load, and there is a lack of time synchronization between perception modules and graphics updates, resulting in high overall latency. More importantly, the dynamic matching relationship between the user’s movement speed and visual continuity is rarely considered, and a motion-adaptive perspective prediction mechanism has not been established. In summary, current methods lack coordination between model lightweighting and system response, lack closed-loop feedback between multimodal perception and graphics rendering, and have insufficient adaptability to highly dynamic interactive scenarios. This paper applies a joint optimization strategy of parameter distillation and channel pruning, combined with sparse hash coding to improve modeling efficiency; designs a multi-sensor fusion posture tracking module based on Kalman filtering to enhance spatial perception stability; by embedding custom shaders and asynchronous rendering pipelines in the Unity engine, an end-to-end driver architecture from implicit expression is built to real-time interaction, systematically solving the compatibility problem between high-fidelity rendering and low-latency response.

## 3. Lightweight Neural Radiation Field Construction and Real-Time Interactive Rendering Architecture Design

### 3.1. Sparse Hash Scene Coding Based on Instant-NGP

**Figure 1:**
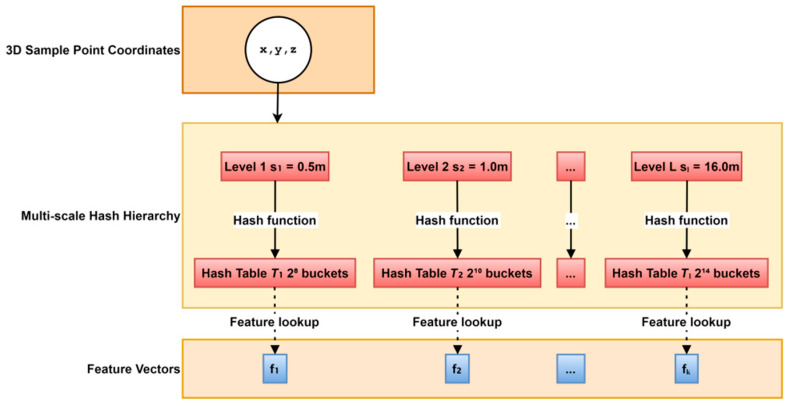
Hierarchical hash coding structure based on the Instant-NGP framework.

Figure 1 illustrates the hierarchical hash coding structure based on the Instant-NGP framework. The system maps three-dimensional spatial coordinates (x, y, z) to corresponding hash tables (T_1_-T_1_) using hash functions of varying scales (h_1_-h_1_). Each level has a spatial granularity increasing from 0.5 m to 16 m and a number of hash buckets ranging from 2^8^ to 2^14^. Each hash bucket stores a 16-dimensional feature vector (f_1_-f_k_). Dynamic training enables adaptive distribution: feature density is enhanced in complex areas such as the surface of an exhibit, while sparse representation is maintained in open areas. Fine-grained levels (e.g., 0.5 m) accurately capture the texture details of the artifact, while coarse-grained levels (e.g., 16 m) maintain the overall structure of the scene. This multi-resolution mechanism can intelligently adjust features according to the observation distance, calling fine levels for close views and coarse levels for distant views, ensuring visual fidelity and optimizing computational efficiency, providing core support for real-time, high-quality rendering in digital devices.

#### 3.1.1. Construction of Hierarchical Hash Coding Structure and Feature Indexing Mechanism

The Instant-NGP framework uses multi-scale hash tables to non-uniformly partition the three-dimensional space. Each layer of the hash table corresponds to a voxel granularity of a specific resolution, forming a hierarchical spatial index structure. After the input multi-view image sequence is calibrated, its corresponding line of sight is sampled in the scene space, and the coordinates of the sampling points are mapped to hash buckets at each level. The spatial division is achieved through a multi-scale hash function, and each layer performs non-uniform discretization on the input coordinates. The formula is as follows:

(1) $$h_{l}\left(x\right)=\left(\left\lfloor \frac{x}{s_{l}}\right\rfloor \mathrm{mod}{\;T}_{i}\right),\;l\;\in \;\{1,\ldots ,L\}$$

x is the coordinate of the sampling point in 3D space; L is the number of hash levels, which is 8; $s_{l}$ is the spatial granularity of the l-th level; and $T_{i}$ is the number of hash table buckets at that level, which is a modulo operation. This function maps continuous coordinates to discrete hash bucket indices, achieving multi-scale spatial partitioning.

Each hash bucket stores a low-dimensional feature vector that characterizes local geometry and appearance attributes. A randomized hash function maps spatial coordinates to buckets, promoting locality: nearby points often collide into the same bucket, which enhances feature consistency in local regions. During the training phase, the network dynamically adjusts the distribution of feature vectors in the hash table so that high-frequency detail areas (such as edges and texture mutations) obtain higher-density feature coverage. In contrast, open areas maintain sparse expression, achieving adaptive spatial representation. The density field gradient drives the sparsity of the hash table during the learning process. Feature updates are activated only at locations where there are significant changes in the radiation field, while the remaining locations remain in the initial state or are directly skipped, greatly reducing invalid calculations. This structure avoids the memory explosion problem of traditional voxel grids while maintaining a high-resolution representation of complex geometry. The feature vector is decoded by a multilayer perceptron (MLP), outputting local density values and view-dependent color values, which form the basic input of the implicit scene function. The hash code’s parallel query mechanism is adapted to the GPU architecture. A single forward inference can complete feature extraction of tens of thousands of sampling points in milliseconds, providing efficient data support for subsequent real-time rendering.

#### 3.1.2. Differentiable Volume Rendering Design and High-Resolution Radiation Field Generation

The volume rendering module receives density and color signals from the hash-coded structure and performs numerical integration along the line of sight to restore the pixel-level image. Each camera ray is sampled using a hybrid strategy: piecewise uniform sampling provides baseline coverage, while importance sampling increases point density near high-gradient regions (e.g., surfaces), thereby preserving sharp boundaries in the rendered image. The density value of the sampling point is converted into volume density through the activation function, and the color value is output by the direction-dependent MLP in combination with the viewing direction, preserving the non-Lambertian property of the material. The integration process uses a weighted summation strategy to accumulate transmittance and emitted light intensity, ultimately generating a single-pixel RGB (Red, Green, Blue) value and depth information. The color synthesis along the camera ray depends on the weighted integral of volume density and transmittance, which is expressed in discrete form as:

(2) $$C\left(r\right)=\sum_{i=1}^{N}T_{i}\left(1-exp\left(-\sigma_{i}\;\delta_{i}\right)\right)c_{i}$$

(3) $$T_{i}=exp\left(-\sum_{j=1}^{i-1}\sigma_{j}\delta_{j}\right)$$

r is the camera ray; $C\left(r\right)$ is the pixel color corresponding to the ray; N is the number of sampling points; $\sigma_{i}$ is the volume density of the *i*-th point; $\delta_{i}$ is the distance from the previous point; $c_{i}$ is the color output by the point; $T_{i}$ is the cumulative transmittance before the light reaches the *i*-th point. This formula enables color synthesis for micro-volume rendering. The depth map is generated by a weighted average of the desired distance to support subsequent geometric reasoning, which is calculated as:

(4) $$D\left(r\right)=\sum_{i=1}^{N}T_{i}\left(1-exp\left(-\sigma_{i}\;\delta_{i}\right)\right)d_{i}$$

$D\left(r\right)$ is the desired depth along the ray, and $d_{i}$ is the distance from the *i*-th sampling point to the camera. This formula is used to generate a per-pixel depth map, supporting subsequent spatial interaction and occlusion detection.

This process is fully differentiable, with gradients backpropagated to hash table features and MLP parameters, enabling end-to-end optimization. The rendering resolution is set to 1920 × 1080 to meet the input requirements of high-definition display devices. To address the surge in computational complexity at high resolutions, the system applies a ray batching mechanism, dividing the image into several tiles for parallel processing, fully leveraging the multi-core parallel capabilities of the graphics card. During training, the L2 loss between the real image and the rendered result drives the model to continuously refine the hash features and network weights, gradually approximating realistic lighting performance. After multiple rounds of iteration, the radiation field accurately restores the original scene’s light and shadow layers, material reflections, and occlusion relationships, creating a visually coherent three-dimensional representation. The generated initial radiation field not only contains a color image with full visual coverage, but also synchronously outputs a pixel-by-pixel depth map, providing geometric priors for subsequent pose estimation and spatial interaction.

**Figure 2:**
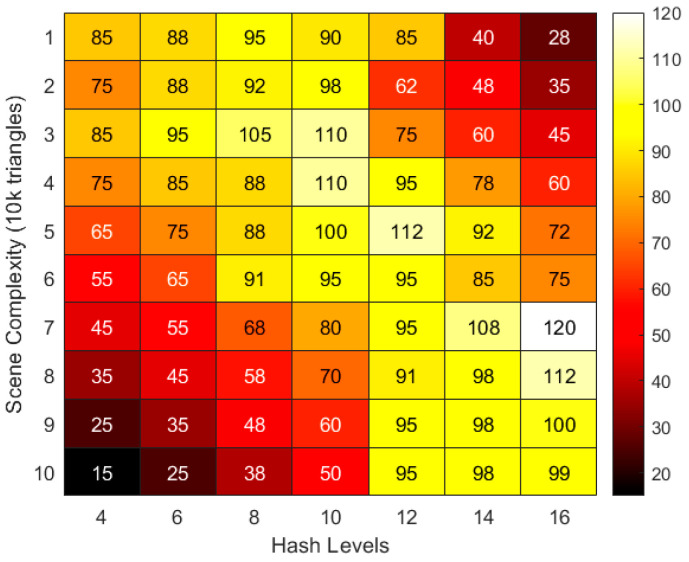
The combined impact of hash level and scene complexity on rendering FPS (Frames Per Second).

Figure 2 shows the combined impact of hashing levels and scene complexity on rendering performance, showing a significant nonlinear coupling. Low-complexity scenes (10,000–30,000 faces) reach peak performance at hashing levels 8–10, attributed to the memory locality optimization and computational load balancing achieved by sparse hashing. However, increasing the hashing level to 14 or higher increases the hash table maintenance overhead, leading to a sharp drop in frame rate. Medium complexity (40k–60k facets) requires 10–12 levels of hashing for optimal matching. Here, spatial granularity and geometric detail density are synergistically optimized, resulting in a peak performance of 112 fps, reflecting maximized feature sampling efficiency. High-complexity scenes (>70k facets) rely on 12–16 levels of hashing to maintain above 90 fps. The data reveals that there is a critical threshold for layer selection: below the threshold, spatial undersampling leads to information loss, while above the threshold, computational redundancy dominates performance degradation. The distribution verifies the adaptive limitations of hierarchical hash coding in Instant-NGP-fixed layer configuration cannot be globally optimal and needs to be dynamically adjusted according to the scene’s geometric density, providing structural optimization direction for subsequent model lightweighting.

### 3.2. Radiation Field Parameter Distillation and Model Lightweight Design

#### 3.2.1. Directed Extraction and Structured Compression Design of Density and Color Weights

After training convergence, the NeRF model contains a set of network parameters that implicitly encode the scene geometry and appearance. Its MLP backbone stores weight tensors for the density and color branches. A layer-by-layer traversal strategy is used to extract these weights, separating the fully connected layer parameters related to volume density prediction from the independent branch parameters for view-dependent color generation. The extraction process focuses on the response features under a key set of viewpoints, which cover typical viewing directions and areas with high texture complexity, ensuring that weight patterns that have a significant impact on visual fidelity are preserved. Channel pruning is performed on the extracted weight matrix to remove neuron channels whose activation responses are lower than a preset threshold in a structured manner. The pruning operation is performed on the filter dimensions in the convolution equivalent structure. The first k channels that contribute most to the output feature map are retained in each layer, and the rest are set to zero and permanently removed. Channels are retained based on the magnitude of their weights. Specifically, a layer-wise threshold determines which channels to preserve, as formalized below:

(5) $$M_{l}=\left\{m|\frac{1}{\mathrm{HWC}}\sum_{h,w,c}\left|W_{l}^{\left(m\right)}\left(h,w,c\right)\right|>\tau_{l}\right\}$$

$W_{l}^{\left(m\right)}$ is the weight tensor of the *m*-th convolution kernel in layer *l*; H, W, and C are its spatial and channel dimensions; $\tau_{l}$ is the pruning threshold for this layer; and $M_{l}$ is the set of retained channel indices. This criterion is used to select highly responsive channels and implement structured pruning.

This process applies a sparse regularization term to constrain the weight distribution in the later stages of training, forcing the network to automatically weaken redundant paths during convergence, improving functional integrity after pruning. The pruned model retains the original network topology, reducing only the number of channels per layer to ensure compatibility with memory alignment requirements for subsequent hardware deployment. The pruning ratio is dynamically adjusted based on the gradient amplitude of each layer. Higher layers near the output retain more channels to maintain color consistency, while lower layers allow for greater compression, achieving non-uniform but semantically sound parameter reduction. During inference, the compressed network skips computations for the pruned channels, significantly reducing the total number of floating-point operations while maintaining the ability to express key geometric boundaries and material transitions.

#### 3.2.2. Low-Activation Neuron Freezing and High-Frequency Weight Protection Mechanism

After completing channel pruning, the activation states of neurons in the remaining network are statistically analyzed. Using multi-view data to drive forward propagation, the response amplitudes and activation frequencies of neurons in each layer are recorded under different input conditions. The statistical results show that some neurons output values close to zero at the vast majority of sampling points, indicating that their contribution to scene representation is minimal. A freezing operation is performed on these low-activation neurons, fixing their outputs to a constant and cutting off their computational path during inference. The freezing operation acts on the activation function input, setting the corresponding weight connections to non-updatable and skipping their matrix multiplication operations during inference, further reducing the computational load. In parallel, it is identified that weight layers are sensitive to high-frequency details, typically located in modules near the color output in the later stages of the network. These layers, which exhibit strong responses to edges, texture details, and sudden changes in illumination during training, are marked as protected layers. The protection mechanism employs a gradient amplification strategy to enhance the loss weights of these layers during the fine-tuning phase, preventing them from being excessively perturbed by pruning and quantization. Furthermore, the weights in the protection layer are excluded from channel culling, allowing only limited numerical compression. This strategy ensures that subtle structures such as engravings, material grain, and specular reflections are accurately restored after compression.

#### 3.2.3. Mixed-Precision Quantization and Lightweight Radiation Field Packaging Design

Mixed-precision quantization is performed on pruned and frozen networks, converting floating-point 32-bit (FP32) weights to integer 8-bit (INT8) or floating-point 16-bit (FP16) formats. This quantization uses a layer-by-layer calibration approach, reconstructing the response distribution from a small subset of viewpoints without relying on additional labeled data, and determining the dynamic range of weights and activations at each layer. The scaling factor is optimized by minimizing the difference between the output before and after quantization, ensuring that the transformed value mapping is as close to the original precision as possible. The color branch is more sensitive to errors and uses FP16 to preserve the dynamic range, while the density branch varies smoothly in most areas and is suitable for INT8 compression. The activation function output is also quantized and, along with the calibrated scaling parameters, restored to FP32 space during inference for subsequent computation. The quantized model parameters are reorganized into a compact binary format, removing redundant metadata and debugging information, retaining only the tensor structure and hash table indexes required for inference. The resulting model is packaged as a lightweight radiation field model that can be loaded onto edge devices and run on embedded GPUs with minimal memory usage. The model is smaller than the original NeRF model, and loading time is compressed to seconds, meeting the needs of rapid field deployment. The entire lightweight process achieves a balance between computational efficiency and visual fidelity without reconstructing the network architecture, providing schedulable implicit scene representation for subsequent real-time interaction. Table 1 shows a comparison of key parameters before and after optimization of the NeRF-based lightweight model.

### 3.3. Multimodal Perception Data Fusion and Spatial Positioning Design

**Figure 3:**
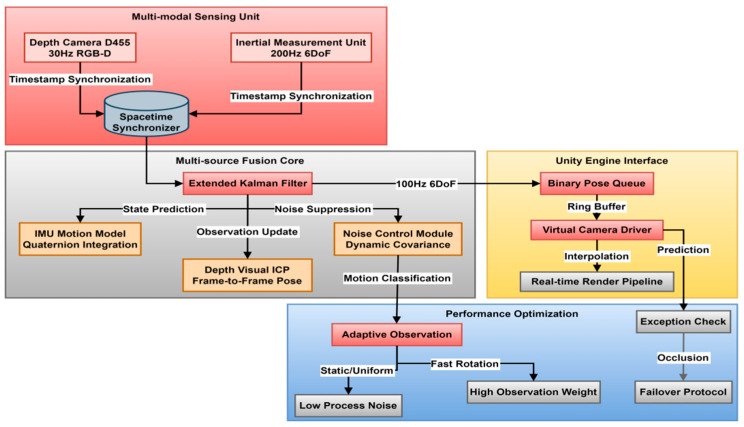
Multimodal pose tracking technology architecture.

Figure 3 illustrates a multimodal pose tracking system for immersive digital devices. The system utilizes hardware-level timestamp synchronization between a depth camera and an inertial measurement unit (IMU) to build a multi-source sensor data fusion pipeline. The core system employs an extended Kalman filter to dynamically balance IMU motion predictions with visual pose observations. Quaternion integration is used to avoid rotational singular values, and a dynamic noise control module is also included. The output provides 6DoF (Six Degrees of Freedom) pose data to the Unity engine at a 100 Hz frequency, ensuring end-to-end latency within 16 ms through a ring buffer and interpolation compensation. The performance optimization module recognizes user motion states (stationary, constant speed, and rapid rotation) in real time, automatically adjusts sensor weights, and handles occlusion anomalies. This perfectly addresses the challenges of multi-person interaction, rapid perspective switching, and complex lighting common in museum scenes, providing a precise spatial positioning foundation for NeRF rendering.

#### 3.3.1. Spatiotemporal Alignment and Joint Filtering Architecture for Multi-Source Sensor Signals

The depth camera and inertial measurement unit are deployed on the same rigid structure, forming a collaborative perception unit. The depth camera outputs dense depth maps and RGB frames at 30 Hz. After internal calibration, the point cloud sequence is generated. Feature matching and the ICP (Iterative Closest Point) algorithm are combined to calculate inter-frame displacements, resulting in high-precision but low-frequency pose increments. The IMU samples angular velocity and linear acceleration at 200 Hz and integrates them to generate high-frequency motion trajectories, which can lead to bias drift and noise accumulation. Since the two types of data are sampled asynchronously on the time axis, a hardware timestamp alignment strategy is used to map the depth frame and IMU measurement sequence to a unified clock domain, interpolating the data to generate a continuous state estimate at the corresponding moment. The spatial coordinate system is determined by external parameter calibration, and the rigid body transformation matrix between the optical center of the depth camera and the center of mass of the IMU is accurately solved in the calibration field to ensure geometric consistency in the subsequent fusion process. The extended Kalman filter constructs a state vector containing position and attitude quaternions, linear velocity, angular velocity, and sensor bias terms. The system dynamics model propagates the state using IMU measurements, with attitude represented as a quaternion to avoid Euler angle singularities in the nonlinear motion equations. Attitude prediction is updated by integrating the quaternion differential equation, which is discretized as:

(6) $$q_{k}=q_{k-1}⊗\left(\frac{1}{2}\Omega \left(\omega_{k}\right)\Delta t\right)$$

$q_{k}$ is the attitude quaternion at time k; $\omega_{k}$ is the angular velocity measured by the IMU; Ω is the quaternion differential operator corresponding to the angular velocity; ⊗ is the quaternion multiplication; and Δt is the integration step size. Formula (6) is used to extend the attitude prediction step in the Kalman filter.

The observation model uses the pose difference output by the depth camera as the external observation and constructs the residual term to correct the IMU integral error. The pose observation residual is calculated by the Lie group transformation difference, and its expression is:

(7) $$z_{k}=T_{\mathrm{depth}}^{-1}\cdot {\hat{T}}_{k}$$

$z_{k}$ is the observation residual; $T_{\mathrm{depth}}^{-1}$ is the pose calculated by the depth camera; and ${\hat{T}}_{k}$ is the current pose predicted by the filter. This residual serves as the observation input, driving the Kalman gain update to achieve pose correction.

The prediction phase uses high-frequency IMU data to continuously update the state. The update phase triggers observation correction when depth frames arrive to suppress integral drift. The filter covariance matrix dynamically adjusts the weights of process noise and observation noise, adaptively adjusting according to motion intensity: reducing process noise gain during stationary or constant speed phases to maintain stability. Observation weights are increased during rapid rotation or acceleration/deceleration to enhance response sensitivity.

#### 3.3.2. Real-Time Transmission and Engine-Driven Mechanism of 6DoF Pose Stream

The pose output by the filter includes both 3D position and orientation expressed as quaternions, forming a complete 6-DOF motion description. This data stream is encapsulated as a binary protocol packet via a serial communication interface and pushed to the host Unity runtime environment via a low-latency transmission channel. The protocol design utilizes a lightweight frame header structure that includes a timestamp, checksum, and pose fields to avoid redundant metadata overhead. An asynchronous listening thread is deployed on the receiving end to handle packet parsing independently of the main rendering loop, preventing network jitter from blocking the main thread. The parsed pose information is stored in a shared memory buffer, and a circular queue is used to manage the latest state, ensuring that each rendering call obtains the pose snapshot closest to the current frame time. The transformation matrix of the virtual camera in Unity is directly driven by this pose, with the position and rotation fields mapped to the Transform component, achieving synchronous mapping of the virtual perspective to the user’s head movement.

To eliminate the slight time offset applied by sampling and transmission, the system implements linear interpolation compensation, using the timestamp difference to smoothly transition between two adjacent valid poses, avoiding image jumps. Interpolation is limited to a 10-ms time window. If the threshold is exceeded, predictive extrapolation is activated, using the velocity and angular velocity of the previous cycle to estimate the current pose and maintain visual continuity. The pose update frequency is stable at 100 Hz, meeting the human eye’s threshold for motion smoothness. In rapidly rotating scenes, the system detects sudden changes in angular velocity and automatically increases interpolation weights to mitigate visual lag caused by delayed sensor response.

### 3.4. Real-Time Rendering Pipeline Integration Design in Unity Engine

#### 3.4.1. GPU-Level Coupling of Custom Shaders and NeRF Inference Interfaces

A full-screen post-processing shader is built into the Unity rendering pipeline. The core computational logic is written in HLSL (High Level Shader Language), embedding implicit field query instructions for the lightweight NeRF model. This shader intervenes in the rendering process before the camera completes rendering of each frame, receiving the virtual camera’s current viewpoint position and viewing direction as input parameters. Pixel coordinates within the frustum are converted back to world-space rays through inverse projection. Each ray is then fed into the NeRF network computation process as a query starting point. The shader internally declares a CUDA (Compute Unified Device Architecture) kernel function interface and calls the precompiled.pt model weight file via a DLL (Dynamic Link Library) link, enabling GPU-directed inference. The CUDA kernel enables direct access to hash tables and compressed network parameters in video memory, eliminating frequent CPU–GPU data transfers. The ray sampling strategy implements adaptive step-size control in the shader, using dense sampling in near-field areas and gradually sparse sampling in distant areas to optimize computing resource allocation.

The sampling point coordinates are mapped to feature vectors using multi-resolution hash encoding and fed into a compressed MLP network for density and color prediction. The network’s forward pass runs entirely on GPU Tensor Cores, leveraging TensorRT to optimize operator fusion and memory reuse, improving computational throughput. The color output undergoes gamma correction and tone mapping before being written to the frame buffer to form the final perspective image. The entire inference process is completed in a single pass, without relying on multi-frame accumulation or history buffering, ensuring that each frame output is independent and complete. Shader supports dynamic switching between different scene models, loading the corresponding CUDA context through texture handle indexing, enabling fast switching between multiple regions. An exception handling mechanism embedded in the kernel call layer automatically downgrades to low-resolution rendering mode when inference timeouts or video memory overflows are detected, maintaining continuous system operation. This shader seamlessly integrates with Unity’s standard rendering process, enabling the overlay of post-production effects such as depth of field and anti-aliasing to maintain a consistent visual style.

#### 3.4.2. Double Buffering Mechanism and Timing Scheduling of Asynchronous Rendering Queues

A double-buffered architecture decouples NeRF rendering from display: while one GPU frame buffer is being written, the other is being scanned out, ensuring tear-free output synchronized with vertical refresh. While a CUDA kernel is writing one buffer to the current frame rendering result, the other is being sent to the display pipeline for output to a projection device or head-mounted display. Buffer switching is triggered by the vertical sync signal, ensuring that image updates are strictly aligned with the screen refresh rate, eliminating tearing. Data isolation between buffers prevents write and read conflicts, and each buffer is bound to an independent CUDA stream, enabling parallel computing and transmission. The asynchronous loading queue, managed by a separate thread, is responsible for prefetching and updating model parameters, hash table data, and pose information. This queue monitors pose change trends and pre-loads scene data for areas the user is about to enter, reducing real-time query latency.

The queue uses a priority scheduling strategy, keeping data blocks in high-frequency areas resident in video memory and automatically offloading them to less active areas to limit peak memory usage. Pose input is injected into the buffer queue at a 100 Hz frequency, and the main rendering loop pulls the latest valid pose at regular intervals. If the queue is empty, the previous frame’s state is used to prevent pauses due to missing data. The rendering scheduler monitors GPU load and frame time, dynamically adjusts the number of sampling points and network layers, and implements a hierarchical load reduction strategy: when the frame time approaches the threshold, the ray sampling density is reduced to maintain the target refresh rate. Tasks are split across multiple CUDA streams, with density field queries, color generation, and image synthesis executed separately in separate streams, leveraging the GPU’s multi-core parallel capabilities to minimize memory latency. Video memory management utilizes a pooling mechanism, pre-allocating fixed-size tensor blocks to avoid frequent requests and releases during runtime. Model weights are loaded as read-only constant memory to improve cache hit rates.

## 4. Immersive Interaction Performance Evaluation

### 4.1. Experimental Data

Experimental data are primarily drawn from real-world immersive digital installations, including a structured conference room (low complexity), an open exhibition hall (medium complexity), and a densely populated exhibition hall (high complexity). Data collection includes multi-view image sequences, synchronizes pose information from the depth camera and IMU, and rendering performance metrics under various lighting conditions (low illumination 100 lux, standard illumination 500 lux, and strong backlight 1000 lux). Parameters are recorded using the controlled variable method and compared with native NeRF, MVS, 3D Gaussian Splatting, Voxel Grid, and Point Cloud methods. User interaction data is obtained by evaluating response latency and visual continuity (SSIM and GME) of multiple users under rapid rotation (angular velocity > 0.8 rad/s) and varying movement speeds (0.5–1.5 m/s). The random errors in the experiment mainly originate from sensor noise and user movement deviations. These are quantified through repeated experiments, with results reported as mean ± standard deviation. System errors, stemming from equipment calibration deviations and clock synchronization issues, are mitigated through hardware calibration and timestamp alignment prior to data acquisition, with compensation implemented in the filtering algorithm. All quantitative indicators were derived from multiple independent replicate experiments, with their distributions meeting the normality assumption through Shapiro–Wilk test (*p* > 0.05) and confirming homogeneity of variance between groups via Levene test (*p* > 0.05). Residual analysis showed no significant autocorrelation (Durbin-Watson test d ≈ 2.0). A one-way ANOVA was then employed to compare multiple groups, ensuring the validity of statistical inferences.

### 4.2. Dynamic Analysis Driven by Sparse Hash Coding

A sequence of 50 uniformly distributed views of the surrounding scene is selected as the input sequence. The spatial distribution of the sampling points corresponding to each view is recorded, and its spatial entropy is calculated to measure information density. The pixel-level difference between the rendered image and the ground-truth image at that view is simultaneously obtained to calculate the L2 error. After training convergence, the gradients of each layer of the MLP network are extracted during each forward-backward propagation process, and their L2 norms are calculated and normalized by viewpoint, reflecting the contribution of each direction to the model update. All data are smoothed to remove high-frequency noise and ensure discernible trends.

**Figure 4:**
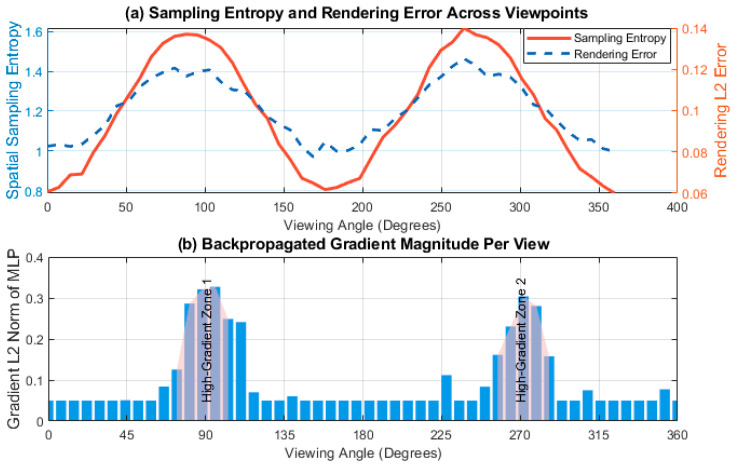
Dynamic analysis of multi-perspective modeling driven by sparse hash coding. (**a**) Sampling Entropy and Rendering Error Across Viewpoints, (**b**) Backpropagated Gradient Magnitude Per View.

Figure 4 systematically reveals the synergistic mechanism between Instant-NGP’s sampling strategy and learning dynamics during multi-view modeling. The figure shows a significant increase in sampling entropy around 90° and 270°, corresponding to observation angles with complex geometric structures in the scene. The system automatically increases spatial sampling density to capture boundaries and curvature changes, thereby maintaining low rendering error. This demonstrates the viewpoint-adaptive information focusing capability of hash coding. The figure below shows that the gradient L2 norm peaks at the same angle, indicating that these perspectives contribute most to the network parameter update during backpropagation. The model prioritizes optimizing the implicit field expression in areas with high information density, verifying that the sparse hash structure guides the sampling distribution through spatial entropy and dynamically concentrates learning resources on key perspectives, forming a closed-loop optimization path of “high entropy input-strong gradient feedback.” This mechanism avoids global uniform computation, significantly improving training efficiency and geometric fidelity, and particularly demonstrating stronger expressiveness in texture-rich or frequently occluded areas. Data fluctuations stem from sampling overlap and noise perturbations caused by viewpoint continuity, but the overall trend remains stable, reflecting the system’s robustness. Instant-NGP does not passively respond to input data, but actively identifies the value of information, drives the sparse learning process, and provides an efficient and accurate modeling foundation for real-time radiation field construction.

It selects multiple sets of feature vector configurations of different dimensions and performs an end-to-end training process on a standard indoor scene dataset. The PSNR (Peak Signal-to-Noise Ratio) value between the reconstructed radiation field image and the real image is recorded every thousand iterations to construct a three-dimensional dataset of iteration quality. It synchronously collects GPU memory access logs, counts the number of hash queries per unit time for each spatial region, classifies regions by structural characteristics, and generates a load distribution matrix after normalization. During training, it maintains fixed sampling strategies and optimizer parameters to ensure variable uniqueness. Visualization tools are used to extract the trend of surface gradient changes, and the rationality of query frequency is verified by combining it with memory bandwidth usage to complete multidimensional performance evaluation.

**Figure 5:**
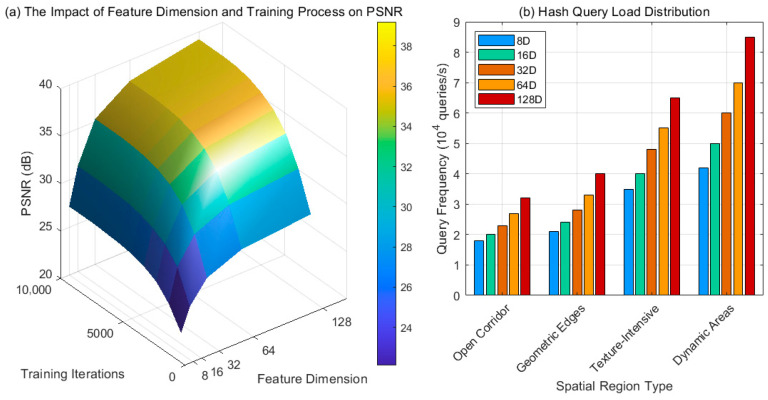
Trade-off analysis between time and space efficiency and quality of sparse hash coding. (**a**) The Impact of Feature Dimension and Training Process on PSNR, (**b**) Hash Query Load Distribution.

Figure 5a illustrates the synergistic effect of feature dimension and training progress on the quality of radiation field reconstruction. The surface exhibits a growing and converging trend, revealing the progressive convergence of hash coding: the initial steep gradient corresponds to the rapid construction of geometric contours, while the saturation at the end indicates that the implicit field has approached the information entropy limit. Low-dimensional features (8D) converge but are limited by their expressive capacity. High-dimensional features (128D) capture subsurface scattering and microgeometric variations through a richer vector space. Their higher slope demonstrates the deep feature mining capabilities of sparse hashing structures. Figure 5b reveals the spatially heterogeneous distribution characteristics of query load. Dynamic areas and texture intensities show significantly higher query density, validating the adaptive sampling mechanism of hash coding: the system automatically focuses computing resources on areas where the radiation field gradient changes dramatically. As feature dimensionality increases (8D–128D), global query frequency increases linearly, but response slopes vary across different spatial types, indicating increased sensitivity of high-dimensional representations to complex geometry. This distribution confirms that sparse hashing optimizes efficiency through uneven resource allocation, maintaining accuracy in critical areas while suppressing computational overhead in inefficient regions, forming an intelligent computing paradigm oriented toward visual perception priorities.

### 4.3. NeRF Lightweight Multidimensional Performance Evaluation

A multidimensional parameter space is constructed covering a continuous range of pruning rates, quantization bit widths, compression ratios, and sparsity coefficients, ensuring sufficient sampling density to capture nonlinear responses. It performs forward inference, configuring each parameter combination and recording raw latency and model volume data, excluding transient values during startup and loading, and taking the average response time under stable operation. Memory usage is double-checked by measuring the model’s serialized file size and runtime memory footprint to eliminate cache interference. The raw data for inference time and memory usage is normalized to the 0–1 range to eliminate dimensionality and enable cross-metric comparison.

**Figure 6:**
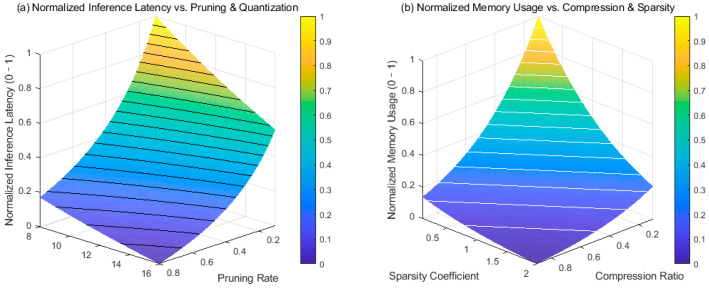
NeRF lightweight multidimensional performance evaluation. (**a**) Normalized Inference Latency vs. Pruning & Quantization, (**b**) Normalized Memory Usage vs. Compression & Sparsity.

Figure 6 reveals how different compression mechanisms affect system performance during NeRF lightweighting. Normalized inference latency decreases nonlinearly with increasing pruning rate, with a steeper gradient within the pruning range. This indicates that channel removal directly reduces the amount of forward MLP computation, significantly reducing the total number of floating-point operations in deep networks. Increasing the quantization bit width further compresses data path bandwidth, but the change is minimal within high pruning rates. The compression ratio and the sparsity coefficient mainly drive the normalized memory usage. Its exponential decay trend stems from the combined effect of parameter pruning and hash table sparsification. The sparsity coefficient has a significant impact on memory in the range of approximately 0.1–1. It tends to saturate after exceeding 1, indicating that the hash structure has an activation lower limit, and excessive sparsity destroys scene connectivity. Optimizing inference latency relies on network structure compression, while memory usage relies more on controlling the total number of parameters. Normalization allows for a consistent trade-off between the two. Data trends indicate that a balance between a pruning rate > 0.6, a compression ratio > 80%, and a sparsity coefficient < 1 is recommended during deployment, maintaining the integrity of the radiation field while meeting edge device resource constraints.

### 4.4. Comparison of Rendering Delay and Frame Rate Under Different Lighting Conditions

The controlled variable method is used to construct three types of lighting environments: low illumination (100 lux), standard light (500 lux), and strong backlight (1000 lux). The light source output is calibrated using a lux meter to ensure uniform lighting distribution within the scene and no flicker interference. The test sequence includes static viewpoints and dynamic roaming paths. Each method continuously renders 300 frames under the same pose trajectory. The time taken to capture a single frame is accurately recorded using GPU timestamps, and the first 50 frames are discarded to eliminate the impact of initialization. Frame rate stability is evaluated using a sliding window with a window length of 60 frames. Our lightweight NeRF approach is compared with native NeRF, MVS, 3D Gaussian Splatting, Voxel Grid, and Point Cloud. Data collection is repeated five times, and the mean and standard deviation are calculated to ensure statistical significance.

**Figure 7:**
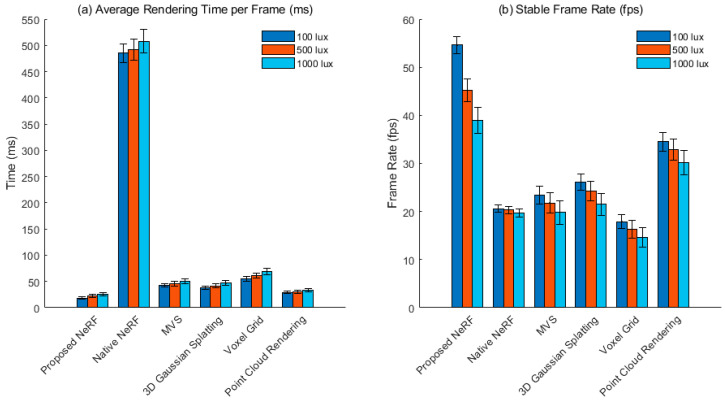
Single frame rendering time and stable frame rate. (**a**) Average Rendering Time per Frame (ms), (**b**) Stable Frame Rate (fps).

Figure 7 compares the performance of six rendering methods in terms of single-frame rendering latency and stable frame rate under different lighting conditions. The lightweight NeRF proposed in this paper maintains the lowest single-frame rendering latency from low illumination to strong backlighting, achieving a rendering latency of 25.7 ms ± 3.4 ms under strong backlighting, significantly outperforming other methods. Native NeRF relies on dense sampling and full-network forward computation, resulting in a high computational load and rendering times far exceeding the real-time interactive threshold. MVS and voxel grids are limited by point cloud density and voxel resolution. Complex lighting conditions require additional lighting compensation and hole repair, increasing processing overhead. While 3D Gaussian Splatting offers the advantage of differentiable rendering, it also suffers from repeated projections caused by covariance matrix distortion in strong backlight conditions, slowing rendering speed. While point cloud rendering is computationally lightweight, it lacks an occlusion inference mechanism and still requires an additional depth synthesis step. Frame rate analysis shows that our method maintains the highest output frame rate across all lighting conditions, achieving a stable frame rate of 39.0 fps ± 2.7 fps in strong backlight conditions. This is due to the coordinated optimization of model compression and asynchronous scheduling. The native NeRF frame rate approaches the real-time lower limit and fluctuates under drastic lighting changes, indicating its sensitivity to input conditions. MVS and Voxel Grid experience significant frame rate drops in strong backlight conditions, attributed to feature extraction failures leading to accumulated pose solution errors. Overall, changes in light intensity have a significant impact on explicit methods. However, the lightweight NeRF proposed in this paper exhibits stronger stability and response consistency in dynamic lighting due to the robust feature indexing of hash coding and the efficient inference of the compressed network, verifying its applicability in real-world deployment scenarios.

### 4.5. Comparison of Model Size and Loading Time Under Different Scene Complexities

Three scenarios are selected: a conference room with a regular structure (low complexity), an exhibition hall with an open layout (medium complexity), and a cultural relic display room with dense furnishings (high complexity). The model file size (MB) and first loading time (s) of the lightweight NeRF method in this paper are compared with those of native NeRF, MVS, 3D Gaussian Splatting, Voxel Grid, and Point Cloud.

**Figure 8:**
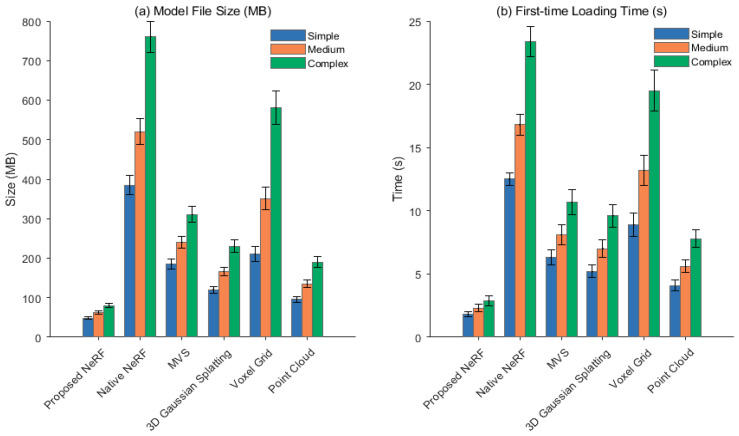
Model volume and loading efficiency. (**a**) Model File Size (MB), (**b**) First-time Loading Time (s).

Figure 8 compares the model size and loading efficiency of six 3D representation methods for varying scene complexity. The lightweight NeRF proposed in this paper maintains minimal storage usage and shortest loading time across all scenarios, and exhibits the slowest increase with geometric complexity. The file size for high-complexity scenes is only 79.5 MB ± 5.3 MB, and the initial loading time is only 2.9 s ± 0.4 s. Due to the high-dimensional hash table and dense MLP parameters of native NeRF, the model size expands nonlinearly with scene detail, resulting in significant input and output pressure during the loading process. MVS relies on storing complete point clouds, resulting in a large amount of uncompressed data. Furthermore, in high-density scenes like cultural relics display rooms, occlusion completion applies redundant points, further exacerbating the storage burden. Voxel grids are limited by resolution, resulting in low spatial utilization. Empty areas still occupy fixed memory cells, leading to a sharp increase in file size. While 3D Gaussian Splatting is explicitly renderable, each Gaussian element contains covariance and spherical harmonic coefficients, resulting in a high parameter density and limited compression efficiency. While lightweight, point cloud methods lack topological compression mechanisms, requiring the reconstruction of index structures during loading. In contrast, our proposed method achieves structural compression through sparse hash coding and channel pruning, retaining only key feature response regions and significantly reducing redundant data storage. Quantized weights and binary packaging optimize read efficiency, maximizing memory bandwidth utilization during loading. This advantage is particularly pronounced in highly complex scenarios, demonstrating its robust adaptability to storage resources and boot response requirements in real-world device deployments.

### 4.6. Comparison of Interactive Response Delay and Jitter Error in Multi-User Pose Tracking

In scenarios supporting 3–5 people interacting simultaneously, the mean and standard deviation of the interaction response delay (ms) and the RMS (Root Mean Square) error (°) of the view jitter of this paper’s lightweight NeRF method, native NeRF, MVS, 3D Gaussian Splatting, Voxel Grid, and Point Cloud Rendering under conditions of rapid view rotation (angular velocity > 0.8 rad/s) are recorded.

Table 2 compares the interactive response latency and view jitter RMS error of six rendering methods in a multi-user concurrent environment during rapid view rotation. The proposed lightweight NeRF performs best in both metrics, with an average interactive response latency of only 16.3 ms, significantly lower than other methods, and an average jitter error of only 0.12°, demonstrating its stability in highly dynamic interactions. Native NeRF, due to dense sampling and full-network inference, results in a latency of up to 482.7 ms, failing to meet real-time requirements. Furthermore, the output sequence exhibits noticeable tearing and prediction lag, leading to significant visual jitter. MVS relies on point cloud reprojection, which can cause pose jumps under fast motion due to feature matching lags, resulting in even higher jitter errors. While 3D Gaussian Splatting offers relatively fast rendering capabilities, untimely covariance updates can lead to viewpoint extrapolation distortion and apply periodic oscillation errors. Voxel grid and point cloud methods are limited by spatial resolution and indexing efficiency, resulting in latency ranging from 58 to 72 ms and moderate jitter. All compared methods exhibit *p*-values less than 0.001 compared to our proposed method, indicating statistically significant performance differences.

### 4.7. Comparison of Visual Continuity and User Movement Speed Adaptability

When the user moves at linear speeds of 0.5 m/s, 1.0 m/s, and 1.5 m/s, the SSIM (Structural Similarity Index) and Gradient Magnitude Entropy (GME) of the lightweight NeRF method in this paper are measured against native NeRF, MVS, 3D Gaussian Splatting, Voxel Grid, and Point Cloud Rendering.

Table 3 compares the visual continuity and detail representation capabilities of the six methods at different linear speeds, using SSIM and gradient magnitude entropy (GME) as quantitative metrics. The proposed lightweight NeRF maintains high structural fidelity from low to high speeds, with a gentle SSIM degradation (0.951 ± 0.016 at 1.5 m/s) and a consistently high GME value (7.68 ± 0.15 at 1.5 m/s), demonstrating its ability to stably restore texture details and edge sharpness under dynamic viewing angles. Native NeRF achieves optimal SSIM at rest or low speeds, but significantly degrades at 1.5 m/s, with a larger drop in GME. This is attributed to inference latency leading to inaccurate viewport extrapolation, causing motion blur and structural distortion. At fast speeds, MVS experiences accumulated depth completion errors, resulting in holes and ghosting, and both SSIM and GME decrease in tandem. While 3D Gaussian Splatting renders quickly, the Gaussian kernel fails to align with the viewpoint at high speeds, resulting in blurred edges, brightness oscillations, and a significant decrease in detail entropy. Voxel and point cloud methods are limited by spatial resolution and sampling density, resulting in low GME baselines and increasingly pronounced texture hopping as speed increases. This method, leveraging asynchronous rendering and pose prediction, effectively compensates for viewpoint shifts caused by motion and maintains spatiotemporal consistency in the image sequence.

### 4.8. Failure Cases and Method Limitations Analysis

While the proposed method performs well in most typical scenarios, performance degradation or visual artifacts were observed under two extreme conditions:

The first occurs in areas with extremely sudden changes in high dynamic range (HDR) illumination. When a user moves instantaneously from a low-light environment (<100 lux) to a strongly backlit area (>2000 lux), slight delays in the auto-exposure responses of the IMU and depth camera can lead to a brief inaccuracy in pose estimation. In this situation, the NeRF rendered viewpoint is slightly misaligned with the true motion trajectory, resulting in noticeable geometric distortion (such as cylindrical bending) at the edges of the image. This issue stems from the physical limitations of the sensor hardware, not the algorithm itself. Experimental data shows that in such transitional scenes, the SSIM decreases and the GME fluctuation increases.

The second case involves the reconstruction of ultra-fine, translucent materials. For objects with complex subsurface scattering properties, such as frosted glass display cases and silk fabrics, the lightweight NeRF model struggles to accurately capture the coupled effects of transmission and diffuse reflection due to the limited MLP capacity and insufficient hashing resolution. This results in uneven gloss or blurred internal structures in the rendered image at certain viewing angles. During lateral motion at 1.5 m/s, the SSIM for silk textures degrades compared to static viewpoints.

These examples reveal two key limitations of current approaches: first, a high reliance on the hardware synchronization accuracy of multimodal sensors; second, a limited ability to represent high-order optical properties at very low model capacity. Future work will explore pose compensation mechanisms based on event cameras and design adaptive hashing resolution strategies for specific materials to further expand the system’s applicability.

## 5. Conclusions

This work presents a deployable NeRF-driven immersive interactive system that jointly optimizes visual fidelity and response latency. Through sparse hash coding and parameter distillation techniques, the model size is significantly compressed and inference efficiency is improved, overcoming the computational bottleneck of implicit field rendering. Multimodal sensor fusion combined with the extended Kalman filter enhances the stability of pose estimation under complex motion and suppresses high-frequency jitter. Experiments show that the architecture has the lowest single-frame rendering time of 25.7 ms ± 3.4 ms under strong backlight, a stable frame rate of 39.0 fps ± 2.7 fps, a file size of 79.5 MB ± 5.3 MB in high-complexity scenes, and a first loading time of 2.9 s ± 0.4 s, which is significantly better than native NeRF and other explicit representation methods. The model volume and loading efficiency meet the needs of rapid on-site deployment, and the interaction delay is controlled within the perception threshold, providing a reusable technical paradigm for digital installations in public spaces and pushing the boundaries of the practical application of implicit neural representation in art performances and immersive narratives.

## Figures and Tables

**Table 1:** Comparison of key parameters of NeRF lightweight model before and after optimization.

Parameter/Module	Original Value	Optimized Value	Compression Rate/Improvement
Model Parameters	5.2 M	0.8 M	84.60%
Weight Precision	FP32	INT8 (Density)	4× Storage Saving
		FP16 (Color)	2× Storage Saving
Hash Table Levels	16	16 (Retained)	-
Hash Buckets	2^24^	2^20^	93.75%
MLP Channels	256	128	50%
Active Neuron Retention	100%	62%	38% Pruned

**Table 2:** Interaction response delay and viewing angle jitter RMS error.

Method	Response Latency (ms)—Mean ± Std	View Jitter RMS Error (°)—Mean ± Std	*p*-Value (vs. Proposed NeRF)
Proposed NeRF	16.3 ± 1.8	0.12 ± 0.03	—
Native NeRF	482.7 ± 28.5	0.85 ± 0.11	0.0003
MVS	64.5 ± 6.2	0.38 ± 0.07	0.0004
3D Gaussian Splatting	52.1 ± 5.4	0.31 ± 0.06	0.0006
Voxel Grid	71.8 ± 7.9	0.44 ± 0.08	0.0002
Point Cloud	58.3 ± 5.7	0.35 ± 0.06	0.00003

Note: Std is the abbreviation of Standard deviation.

**Table 3:** Visual continuity and detail expression ability.

Method	Metric	0.5 m/s	1.0 m/s	1.5 m/s
Proposed NeRF	SSIM	0.963 ± 0.012	0.958 ± 0.014	0.951 ± 0.016
	GME	7.82 ± 0.11	7.76 ± 0.13	7.68 ± 0.15
Native NeRF	SSIM	0.971 ± 0.009	0.942 ± 0.018	0.915 ± 0.024
	GME	7.95 ± 0.09	7.52 ± 0.16	7.21 ± 0.21
MVS	SSIM	0.932 ± 0.015	0.901 ± 0.019	0.863 ± 0.023
	GME	7.41 ± 0.12	7.15 ± 0.14	6.83 ± 0.17
3D Gaussian Splatting	SSIM	0.948 ± 0.013	0.935 ± 0.015	0.918 ± 0.018
	GME	7.58 ± 0.10	7.42 ± 0.12	7.25 ± 0.14
Voxel Grid	SSIM	0.921 ± 0.017	0.893 ± 0.019	0.852 ± 0.022
	GME	7.30 ± 0.13	7.05 ± 0.15	6.71 ± 0.16
Point Cloud	SSIM	0.915 ± 0.018	0.887 ± 0.020	0.841 ± 0.024
	GME	7.24 ± 0.14	6.98 ± 0.16	6.63 ± 0.18

## Data Availability

The original contributions presented in this study are included in the article. Further inquiries can be directed to the corresponding author.
